# BCG Vaccination Potentially Modulates the Transcriptome of Infant CD4 T Cells in Addition to Age-Dependent Immune Ontogeny-Associated Changes

**DOI:** 10.3390/vaccines13070706

**Published:** 2025-06-29

**Authors:** Vidya Vijayan Karuvan Kandiyil, Eunchong Kang, Emily Coates, Portia Kamthunzi, Gerald Tegha, Mina Hosseinipour, Di Wu, Fei Zou, Kristina De Paris

**Affiliations:** 1 Department of Microbiology and Immunology, School of Medicine, University of North Carolina, Chapel Hill, NC 27599, USA; vidyakk@email.unc.edu; 2 Department of Biostatistics, Gillings School of Global Public Health, University of North Carolina, Chapel Hill, NC 27599, USA; eunchong@email.unc.edu (E.K.); did@email.unc.edu (D.W.); feizou@email.unc.edu (F.Z.); 3 Vaccine Research Center, National Institutes of Allergy and Infectious Diseases, Bethesda, MA 20892, USA; emily.coates@nih.gov; 4 UNC Project Malawi, Lilongwe P.O. Box 1056, Malawi; pkamthunzi@unclilongwe.org (P.K.); gtegha@unclilongwe.org (G.T.); mina_hosseinipour@med.unc.edu (M.H.); 5 Institute for Global Health and Infectious Diseases, School of Medicine, University of North Carolina, Chapel Hill, NC 27599, USA

**Keywords:** infant CD4 T cell transcriptome, immune ontogeny, BCG vaccination, signaling pathways

## Abstract

Background: The Bacille Calmette–Guérin (BCG) vaccine is part of the Extended Programme on Immunization (EPI) and as such is generally administered at birth. The global introduction of BCG not only protected many vaccinated infants against severe complications of tuberculosis but also resulted in markedly reduced overall childhood mortality. Studies in human adults determined that BCG vaccination induces epigenetic reprogramming of innate immune cells (also known as trained immunity) and can also enhance T cell responses to both mycobacterial and non-mycobacterial antigens. Goal and Methods: The current study tested the hypothesis that BCG immunization similarly impacts the functionally distinct infant immune system. Towards this goal, we applied RNA sequencing to assess transcriptome changes in circulating CD4^+^ T cells of Malawian infants prior to and 2 to 13 weeks after BCG immunization. Results: In the first three months of life, transcriptome changes of infant CD4 T cells implied a functional shift towards T helper 1 and Th17 immunity. Vaccination with BCG resulted in additional modulation of the CD4 T cell transcriptome and differentially expressed genes could be linked to metabolomic function. Conclusions: These findings are consistent with data reported in BCG vaccinated adults and contribute to the understanding of molecular changes in infant CD4 T cells that may explain the improved capacity of the infant immune system to respond to pathogens after BCG vaccination.

## 1. Introduction

Globally, the Bacille Calmette–Guérin (BCG) vaccine is one of the oldest and most widely used vaccines [1]. It is administered to >80% of newborns worldwide and has been shown to prevent severe tuberculosis (TB) complications in infants [2]. Furthermore, the introduction of BCG resulted in a reduction in overall childhood mortality [3,4,5,6]. For example, in Guinea Bissau, a country with a high childhood mortality rate, BCG vaccination was linked to protection against malaria and other unclassified fevers [7]. More recently, severe SARS-CoV-2 disease burden was lowered by BCG vaccination of healthcare workers [8,9].

Agnostic immunity to non-tuberculosis pathogens, including viruses, by BCG vaccination is thought to be mediated by two immunological processes, trained immunity and heterologous T cell immunity. Trained immunity refers to enhanced functional responsiveness of innate myeloid and natural killer (NK) cells in an antigen non-specific manner against various pathogens [10,11,12]. The underlying mechanisms of trained immunity include epigenetic reprogramming, regulation by long non-coding RNAs (lncRNAs), metabolic reprogramming, and increased expression of pathogen recognition receptors (PRRs) [12,13,14,15]. Moreover, ex vivo stimulation of PBMC, or monocytes in particular, with mycobacterial and non-mycobacterial stimuli in BCG-vaccinated adults induced higher cytokine secretion, including IFN-gamma (IFN-γ), tumor necrosis factor alpha (TNF-α), and interleukin-1beta (IL-1β), compared to non-vaccinated controls [12,16,17]. This cytokine response was also accompanied by genome-wide changes in histone modification, in particular H3K27ac, and enrichment of H3K4me3 at the TNF and IL6 promoters, that was maintained up to 1 year after BCG vaccination. Recently, (long-term) transcriptional changes in hematopoietic stem cells and progenitor cells (HSPCs) after BCG vaccination have been demonstrated to confer epigenetic changes in hematopoietic progenitors of peripheral monocytes [5].

While most studies so far have focused on enhanced innate response to BCG, there is evidence that heterologous immunity is also be mediated by T cells. In fact, CD4 and CD8 memory T cells—induced by BCG vaccination—respond to secondary infections with pathogens other than *Mycobacterium tuberculosis* (*Mtb*) by increased proliferation and cytokine production, with CD4 T cells exhibiting T helper 1 (Th1) polarization [12,18]. In infants, a shift towards Th1 immunity might be especially beneficial. Compared to adults, neonates and infants generally lack adaptive immune memory due to the lack of antigen exposure in utero. Moreover, the colonization with commensal microbiota is generally associated with a more Th2-biased immune response to pathogens [19]. Therefore, infants are highly susceptible to many infectious diseases, and those are often associated with higher morbidity and mortality compared to adults [20,21,22,23]. As mentioned above, BCG vaccination of human infants could indeed be associated with protection against malaria [7]. In mice, protection against vaccinia virus and murine malaria by BCG vaccination was demonstrated to be conferred by IFN-γ-producing CD4 T cells [24]. Although the BCG vaccine is routinely administered to infants, there is a gap in understanding the impact of BCG vaccination on the neonatal immune system. To test whether BCG immunization could potentially modulate CD4 T cell function, we performed RNA sequencing in circulating CD4 T cells of Malawian infants prior to and 2 to 13 weeks after BCG vaccination.

## 2. Materials and Methods

### 2.1. Infant Study Subjects and Sample Collection

The study was conducted with infant peripheral blood samples collected at the Area 18 Health Centre in Lilongwe, Malawi, by UNC Project Malawi after obtaining approval by the Institutional Review Board (IRB) at University of North Carolina Chapel Hill and the IRB of the UNC Project Malawi. Institutional guidelines adhere to the World Medical Association’s Declaration of Helsinki. No samples were collected without parental consent. EDTA blood was collected from n = 26 infants at the time of BCG vaccination and 2 to 13 post-BCG vaccination when the infants returned for routine pediatric visits. BCG vaccination at birth or first contact is part of the routine pediatric immunization program in Malawi. The complete list of study subjects with the age at the time of BCG vaccination and blood collection is listed in Table 1. PBMCs were separated by Ficoll-Hypaque gradient centrifugation as described [25] and stored as viable PBMC in liquid nitrogen.

### 2.2. PBMC Isolation

PBMCs were separated by Ficoll-Hypaque gradient centrifugation as described [25]. Briefly, whole blood was diluted 1:2 with PBS and layered over Ficoll-Hypaque and then centrifuged for 30 min without a break at 400× *g*. The white blood cell layer was harvested and washed twice with PBS before counting of PBMC. Note that due to the age of the infants, blood volumes (1 to 3 mL) could be collected. PBMC counts ranged from 2 × 10^5^ to 6.4 × 10^6^, with an average of 2.4 × 10^6^ PBMC (SD: 1.4 × 10^6^). Viable PBMCs (1 vial per infant and per visit) were stored in 90% DMSO/10% FBS in liquid nitrogen until use.

### 2.3. CD4 T Cell and Monocyte Isolation by Fluorescence-Activated Cell Sorting (FACS)

**Figure 1:**
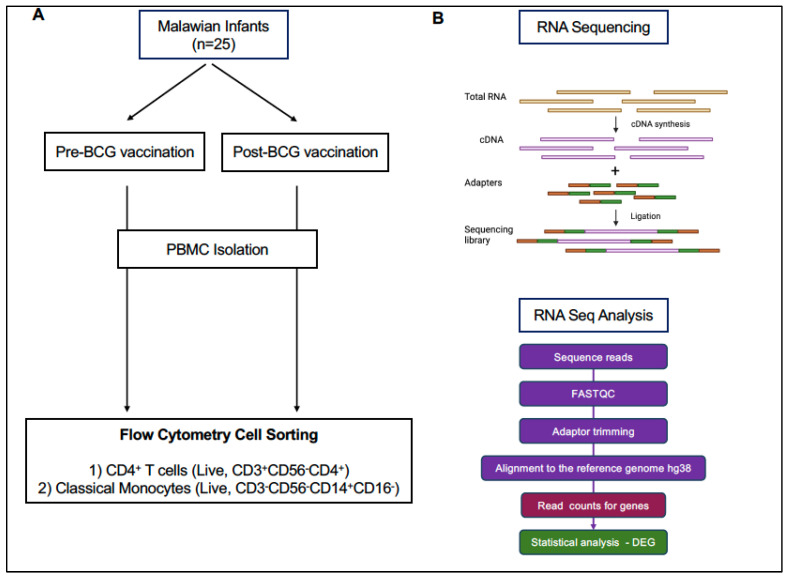
Study Workflow. Panel (**A**): Peripheral blood samples were obtained from Malawian infants just prior to and 2 to 13 weeks post BCG vaccination. PBMC were cryopreserved until live cell sorting for CD4 T cells and monocytes. Sorted cell populations were processed for RNA sequencing. Panel (**B**): Schematic workflows for RNA sequencing and data analysis.

Flow cytometry staining of peripheral blood mononuclear cells was carried out as previously described [25,26,27]. Briefly, PBMC were quickly thawed in a 37 °C waterbath, immediately resuspended in warm RPMI1640 supplemented with 10% FBS, and then washed twice; the second wash was performed with PBS. PBMC were resuspended in 50 mcl PBS, incubated with LIVE/DEAD aqua amine dye (Invitrogen) and then stained for 20 min in the dark with anti-CD3, CD4, CD14, CD16, CD20, and CD56 antibodies (Supplementary Table S1). Next, cells were washed twice with PBS containing 0.1% bovine serum albumin (Sigma Aldrich Inc., St. Louis, MO, USA) and resuspended in 300 μL RPMI-1640 media (Corning Life Science, Glendale, AZ, USA) with 10% FBS (Gibco, Grand Island, NY, USA) and 1% Penicillin-Streptomycin (Gibco, Grand Island, NY, USA) (cRPMI). Cell sorting was carried out by the UNC Flow Cytometry Core Facility on a FACS Aria II (BD Biosciences, San Jose, USA) under BSL2+ conditions. Single, live cells were sorted for CD4 T cells (CD3^+^CD4^+^CD14^−^CD16^−^CD20^−^CD56^−^) and monocytes (CD14^+^CD16^−^CD3^−^CD20^−^CD56^−^) into polypropylene tubes containing 300 μL cRPMI (Figure 1).

### 2.4. RNA Isolation, Library Preparation, and RNA Sequencing

Total RNA was extracted from sorted CD4 T cells and monocytes using the Quick RNA Microprep kit (Zymo Research, Irvine, CA, USA) according to the manufacturer’s instructions. RNA concentration and quality were assessed using a Qubit fluorometer (Life Technologies, Carlsbad, CA, USA) and TapeStation 2200 system (Agilent Technologies, Santa Clara, CA, USA). Libraries were prepared using the Ovation Solo RNA sequence system (NuGen Technology, Redwood City, CA, USA) and RNA sequencing was performed using the Illumina MiSeq 2 × 150 bp paired-end sequencing platform (Figure 1).

### 2.5. RNA Sequencing Data Analysis

Raw FASTAQ files after RNA sequencing were processed using the CLC Genomics Workbench 22.0 (CLC Bio, QIAGEN, Aarhus, Denmark). The raw reads were quality controlled using FASTQC [28] and trimmed for universal and small RNA adaptors, and subsequently mapped and aligned to the hg38 reference genome using the built-in RNA-seq workflow in CLC genomics with default settings (Figure 1). For downstream analysis, gene expression levels were represented by the total number of reads mapped to the exons of each gene (total counts).

To identify differentially expressed genes (DEGs) between pre- and post- BCG vaccination samples in CD4 T cells, the “edgeR” (version 4.0.16) [29] and “limma” (version 3.58.1) [30] packages in R were used to fit a mixed-effects model. First, we used the “cpm” function in edgeR to retain only genes expressed in at least 10% of samples, and counts were then normalized using the trimmed mean of M values (TMM) method via “calcNormFactors” function in edgeR. The normalized data were subsequently processed with two rounds of the “voom” and “duplicateCorrelation” functions in the limma package. Differential Expression (DE) analysis was performed using the “lmFit” and “eBayes” functions in the limma package, to compare gene expression between visits by including a binary visit indicator (0 = pre-BCG, 1 = post-BCG) as the primary comparison. In addition, age at BCG vaccination was considered as a covariate and the subject-level random intercept was included in this mixed-effects model by using lmFit. Estimated Log_2_ fold-changes (FCs) and *p*-values were output. In this model, log fold-change represents the log_2_ difference in gene expression between pre- and post- vaccination. DEGs were defined as genes with an unadjusted *p*-value *p* ≤ 0.05 and an absolute log_2_ FC ≥ 1.

For sensitivity analysis, the same mixed-effects model was fitted, substituting the visit indicator with the “interval” variable as the primary predictor. The “interval” variable was defined as the number of days between the two blood collections, accommodating the varying intervals among subjects and assuming a linear relationship between the vaccine’s effect on gene expression over time. DEGs were identified using an absolute daily log_2_ FC threshold of 0.0159 and an unadjusted *p*-value ≤ 0.05. This daily threshold corresponds to a one-unit increase in log_2_ FC over a median interval of 63 days (i.e., 1 divided by 63), allowing comparison with the threshold used in the primary model.

To assess the effect of age on gene expression, only pre-vaccination samples were used in a fixed-effects model with age at BCG as the primary predictor, without applying the “duplicateCorrelation” function. In this age-effect analysis, DEGs were identified using unadjusted *p* ≤ 0.05 and a log_2_ FC threshold of 0.0159.

For monocyte samples, due to limited paired pre- and post-vaccination data, a fixed-effects model was applied, with the visit indicator as the primary predictor and age at BCG as a covariate. DEG identification criteria were the same as those applied to CD4 T cell analysis.

DEGs were uploaded into Enrichr, a publicly available database that provides access to various gene-set libraries [31,32,33]. We applied KEGG pathway functional annotations and considered them as enriched if their value was lower than 0.05 and ranked them according to their Combined Score (CS), which is a combination of the *p* value and z-score.

Network analyses for interactions of proteins encoded by DEGs were performed using the Search Tool for the Retrieval of Interacting Genes/Proteins (STRING) database, version 11.5 [34,35,36,37,38], which curates both experimental and predicted protein interactions. Protein–protein interactions were visualized using NetworkAnalyst 3.0 [39,40,41,42,43], an open-source software, that utilizes the Human Interactome of the STRING v11.5 database [34,35,36,37,38].

The potential function of long non-coding (lnc) RNAs was examined using the LncSEA 2.0 software [44].

Throughout the manuscript, *p* values refer to unadjusted *p* values if not reported otherwise.

## 3. Results

### 3.1. Study Design, Sample Collection, and Data Analysis

In the original study examining trained immunity in BCG vaccinated adults, PBMC samples were tested at 2 weeks, 3 months, and 12 months following vaccination [14]. In the current study, we selected the 3-month post-BCG time point to allow the identification of transcriptome changes related to normal postnatal immune ontogeny and those potentially indicative of trained and heterologous immunity.

In Malawi, the BCG vaccine is generally administered shortly after birth, prior to the mother and infant leaving the hospital, or at the first infant health visit afterwards. Among the study participants, three infants were vaccinated at day 1 of life, seven infants received the vaccine between day 6 to day 9 of life, ten infants were vaccinated between days 10 to 18, and six infants received the BCG vaccine between one and three months of age (day 31 to day 74; Table 1). A first blood sample was collected just prior to BCG immunization. Most study participants returned within a 2- to 3-month time frame, with a median interval of 63 days between the time of BCG vaccination and collection of the second blood sample, although a few samples were collected as early as 10 days following vaccination (Table 1).

To account for the correlation of repeated measures within individuals and variability in the age of the infant at the time of BCG vaccination, we applied a random effect *limma* approach to probe the transcriptome for DEGs. To identify transcriptome changes that could potentially be associated BCG vaccination, we performed a stepwise analysis. First, as the infant immune system undergoes significant functional changes after birth, we determined age-related changes in gene expression using only the samples collected prior to BCG vaccination. Next, to determine if BCG vaccination was associated with altered mRNA expression, the transcriptome of the samples collected at Visit 2 were compared to the transcriptome at Visit 1, while accounting for age at BCG vaccination. DEGs were defined as having a two-fold change (FC) in mRNA expression (log_2_ FC = 1) and *p* ≤ 0.05. Finally, we conducted a sensitivity analysis using the continuous interval variable to compare DEGs identified by the binary visit indicator model.

### 3.2. Age-Dependent Changes in the Transcriptome of CD4 T Cells

**Figure 2:**
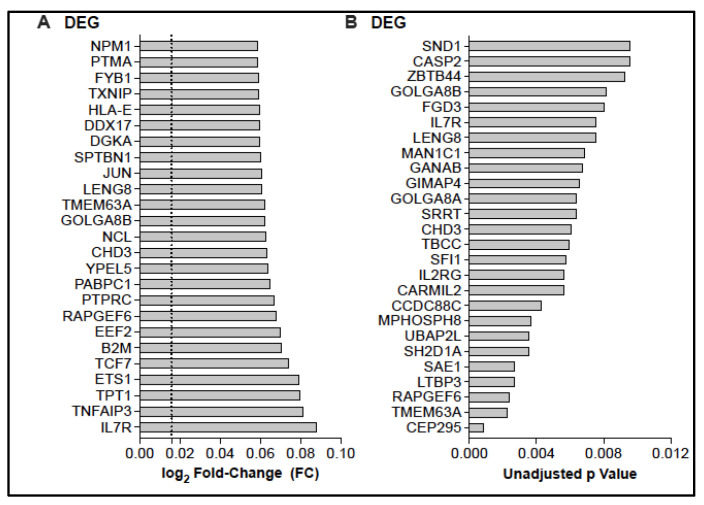
Age-dependent differentially expressed genes. The top 25 DEGs that had increased mRNA expression levels by log_2_ FC (**A**) or listed by *p* value (**B**). The dashed line in panel (**A**) indicates the DEG cutoff of log_2_ FC = 0.0159.

As mentioned above, the infants in the current study were between 1 day and 74 days old when they received the BCG vaccine and the median interval between the first and second blood collection was 63 days. Considering that age is a continuous variable, we rationalized that DEGs dependent on age should have a log_2_ FC = 1 after 63 days and, thus, applied a cutoff of 1/63 or log_2_ FC = 0.0159, with *p* ≤ 0.05. For this analysis, a total of 20 samples were included. Likely due to the small sample size, DEGs could not be assigned when the *p* value was adjusted for multiple comparisons using the Benjamini–Hochberg procedure. After removal of annotated ribosomal genes (e.g., RBM, RPL, RPS, RNF), increased mRNA expression was observed for 377 genes, and down-regulation of mRNA was detected for 2350 genes (Figure 2, Table S2).

**Figure 3:**
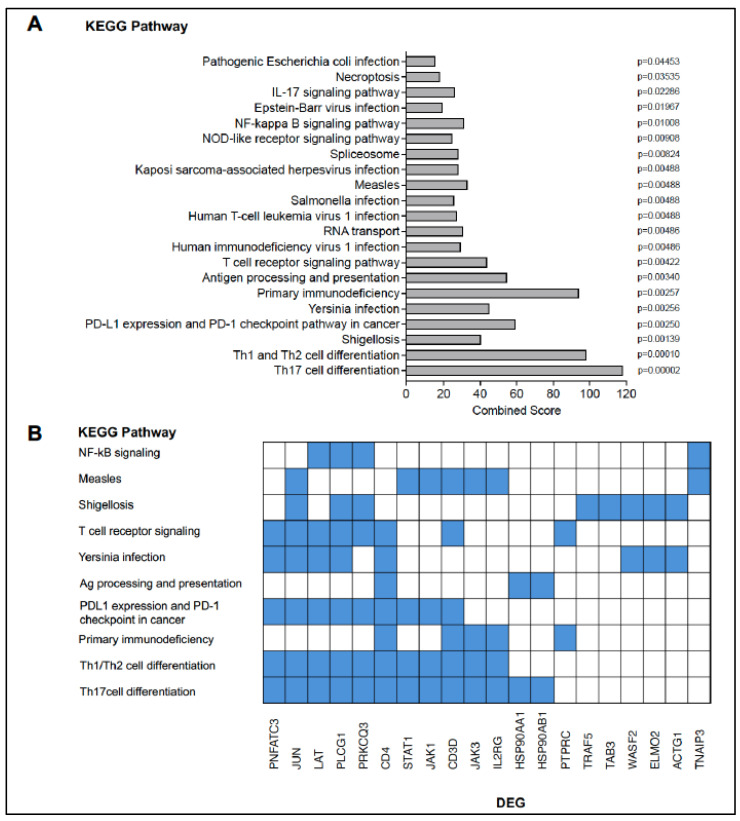
KEGG pathways enriched by DEGs that increased in an age-dependent manner. DEGs that were upregulated with age were uploaded to Enrichr. Panel (**A**) list the KEGG pathways that had an adjusted *p* ≤ 0.5. The specific *p* value for each pathway is listed next to each bar and the enrichment score is listed on the x-axis. Panel (**B**) depicts The clustergrammer of DEGs (bottom axis) enriched (blue fill) in specific pathways (y-axis).

Enrichr analysis revealed that increased DEGs were representative of 21 KEGG pathways with *p* ≤ 0.05 adjusted for multiple comparisons (or 44 pathways if an unadjusted *p* ≤ 0.05 was applied). The top two KEGG pathways corresponded to the T helper 17 (Th17) and Th1/Th2 cell differentiation pathways (Figure 3A). Consistent with this finding, DEGs representative of these specific pathways (Figure 3B) included kinases (JUN, JAK1, JAK3) and transcription factors (NFATC3, STAT1) important in T cell receptor signaling, T cell differentiation, and cytokine signaling.

### 3.3. Potential Changes in the CD4 T Cell Transcriptome Associated with BCG Vaccination

**Figure 4:**
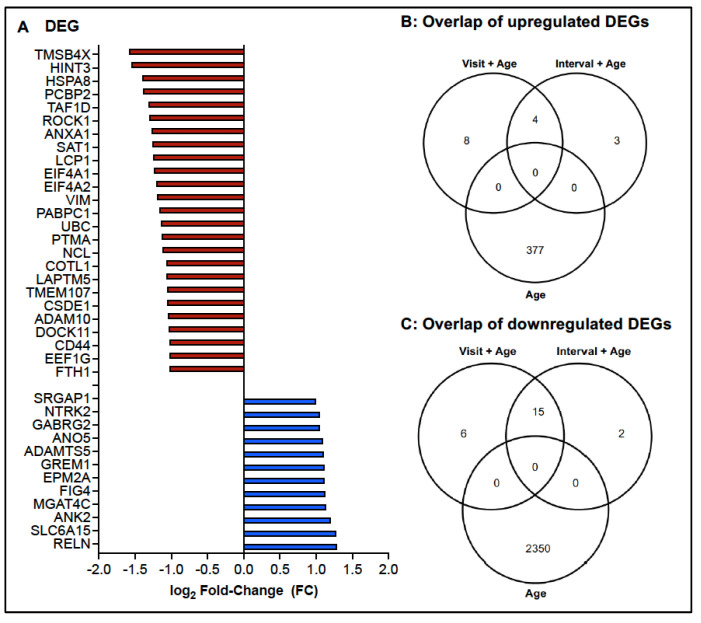
Differentially expressed genes after BCG vaccination. (**A**) Depicted are the 12 DEGs with increased mRNA expression (blue bars) and the top 25 downregulated DEGs (red bars). DEGs were defined as log_2_ FC = 1 and *p* ≤ 0.05, with age at the time of BCG vaccination being considered as covariate. (**B**) Venn diagrams illustrating the overlap of upregulated (**B**) or downregulated (**C**) DEGs (i) applying the age effect model, (ii) the mixed effect model using visit plus age at BCG vaccination (primary analysis), and (iii) the interval effect model (sensitivity analysis).

Based on the findings that age at BCG vaccination was an important confounder of CD4 mRNA expression levels, we tested whether BCG vaccination resulted in altered mRNA expression by comparing transcriptome data at Visit 2 to those of Visit 1, while controlling for age at the time of BCG vaccination. We analyzed 44 samples (22 from Visit 1 and 24 from Visit 2) of 26 infants. Applying DEG criteria of log_2_ FC = 1 and *p* ≤ 0.05, 12 genes were upregulated at Visit 2 compared to Visit 1 and 21 DEGs were downregulated (Figure 4A). There was no overlap of upregulated DEGs in response to BCG vaccination with DEGs that had an age-dependent increase (Figure 4B). Setting DEG criteria to log_2_ FC = 0.58 and *p* ≤ 0.05, 415 genes had increased mRNA levels and 225 DEGs had lower mRNA levels at Visit 2 compared to Visit 1 (Table S3). Even of the 415 upregulated genes, only 2 (ANAPC1 [anaphase promoting complex subunit 1], TBCC [tubulin folding cofactor 1]) overlapped with DEGs identified as age-dependent, further implying a modulation of gene expression by BCG.

The sensitivity analysis using the continuous interval as the primary predictor showed that most DEGs identified by this approach overlapped with those from the primary analysis that considered visit number as well as age at the time of BCG vaccination. Therefore, the variability in the interval between visits among subjects likely had minimal influence on DEG identification. Specifically, of the 24 DEGs (n = 7 with increased mRNA and n = 17 with decreased mRNA; Table S4) identified by the sensitivity analysis, 19 were also present in the primary analysis results (Figure 4B,C). We selected the binary visit indicator model as the primary model, considering the assumption of a linear effect of vaccination over time on gene expression might not be appropriate.

**Figure 5:**
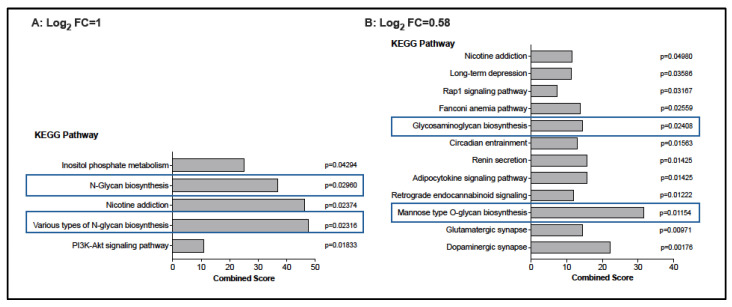
KEGG pathways implicated in BCG-associated DEG induction. Panels (**A**,**B**) list the KEGG pathways that were significantly enriched (unadjusted ps0.05) in DEGs induced by BCG vaccination when a cutoff of log_2_ FC = 1 (**Panel A**) or log_2_ FC = 0.158 (**Panel B**), respectively, was applied. Pathways associated with glycan biosynthesis are highlighted by blue borders.

To probe the potential function of these genes, we subjected the upregulated DEGs to Enrichr analysis. Applying the DEG cutoff of log_2_ FC = 1, 5 KEGG pathways were identified, compared to 12 pathways that represented DEGs with log_2_ FC ≥ 0.58 (all pathways with *p* ≤ 0.05). Independent of the log_2_ FC cutoff for DEGs, KEGG pathways associated with glycan biosynthesis emerged as significant (Figure 5). In addition, several pathways indicative of interactions between the immune function of CD4 T cells and neuromodulatory function (dopaminergic synapse, glutamergic synapse, nicotine addiction) were identified.

**Figure 6:**
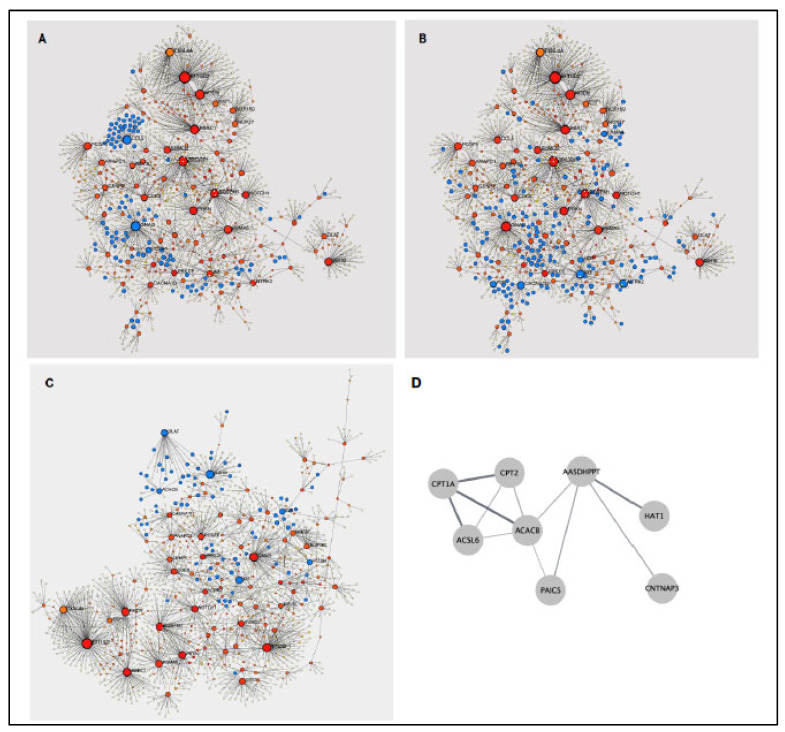
Suggested networks of DEG-encoded proteins and their involvement in KEGG pathways. DEGs upregulated after BCG vaccination were analyst in NetworkAnalyst for the interaction of the DEG-encoded proteins and tested for KEGG pathway modulation. Panel (**A**): Protein-protein interaction network with genes involved in chemokine signaling indicated in blue. Panel (**B**): Blue nodes indicated proteins that are part of the MAPK or PI3K-Akt signaling pathways. Panel (**C**): Examples of DEG-encoded proteins that are involved in cell metabolism (blue), such as The citrate cycle, glucagon synthesis, or phospholipase D signaling. Panel (**D**): A small subset of proteins was implicated in fatty acid metabolism.

The analysis of potential protein interactions of upregulated genes by NetworkAnalyst revealed that DEG-encoded proteins might impact over 100 KEGG pathways (Table S5). The top modulated KEGG pathway (*p* = 1.72 × 10^−37^; false discovery rate [FDR] *p* = 5.48 × 10^−35^) was the chemokine signaling pathway, mediated via the node CCL5 (Figure 6A). Several proteins were involved in T cell signaling, including the mitogen-activated protein kinase (MAPK) and phosphatidylinositol-3-kinase (PI3K)-AKT KEGG pathways (Figure 6B). In addition, pathways associated with metabolic function (Figure 6C) were identified. Among those were DEG-encoded proteins included in the citrate cycle (dihydrolipoamide S-acetyltransferase [DLAT] node), in glucagon synthesis, and in fatty acid metabolism (Figure 6D). 

Long non-coding RNAs (lncRNAs) have emerged as essential factors in gene regulation, such as chromatin modification, activation or inhibition of transcription, mRNA stability, and post-translational modifications [45]. In CD4 T cells, 189 lncRNAs were upregulated in response to BCG vaccination when the less stringent cutoff of log_2_ FC ≥ 0.58 and *p* ≤ 0.05 was applied (Table S6). Several of these lncRNAs were implicated in histone modifications (Table 2), indicative of BCG-associated epigenetic modifications.

In summary, ex vivo analysis of the transcriptome of Malawian infants post-BCG vaccination compared to pre-BCG vaccination revealed changes in the expression levels of genes and regulatory lncRNAs that were indicative of immune activation and increased metabolic function. Furthermore, the induction of these DEGs in response to BCG could not be solely ascribed to immune ontogeny.

### 3.4. Transcriptome Changes in Infant Peripheral Blood Monocytes

**Figure 7:**
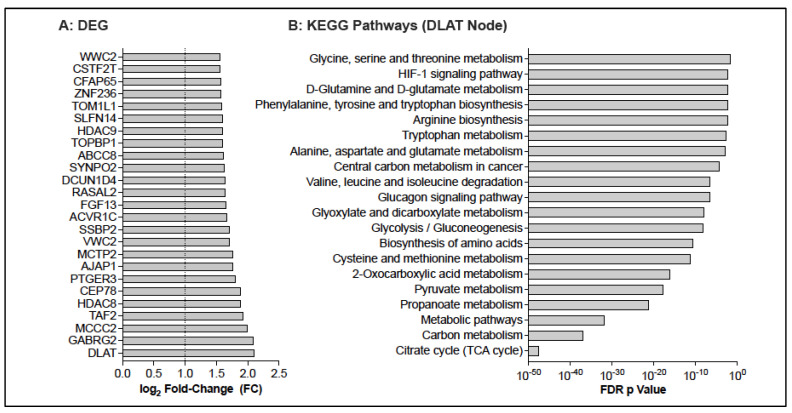
Differentially expressed genes in monocytes of BCG vaccinated infants. Panel (**A**): The top 25 up-regulated DEGs at Visit 2 compared to Visit 1. Panel (**B**): The analysis of DEG-encoded proteins in NetworkAnalyst identified an enrichment of metabolomic pathways associated with the DLAT node network.

The current study was performed when our team had no access to single-cell RNA sequencing. Due to the small blood volumes of infants, we had to pool CD3-CD14+ monocytes from several infants and performed differential mRNA expression analysis of 13 monocyte samples (5 from Visit 1 and 8 from Visit 2) of 12 infants. Changes in monocyte mRNA levels at Visit 2 were estimated using a fixed-effects model with adjusting for visit plus age at BCG vaccination. A total of 362 increased DEG (log_2_ FC = 1, *p* ≤ 0.05) were increased at Visit 2 compared to pre-vaccination, and 130 genes were downregulated (Figure 7A; Table S7). DLAT emerged as the gene that was most highly increased in response to BCG. Consistent with this finding, the citrate cycle emerged as a top KEGG pathway when the upregulated DEGs were analyzed in Enrichr (unadjusted *p* = 1.85 × 10^−4^; adjusted *p* = 0.0410; combined score = 94.41). In NetworkAnalyst, a subnetwork of 36 DEG-encoded proteins—with DLAT as key node—was identified that was representative of several metabolic KEGG pathways, including the citrate cycle, pyruvate metabolism, glycolysis/gluconeogenesis, and fatty acid biosynthesis (Figure 7B), pathways previously identified as key mediators of trained immunity [13].

Among the top upregulated genes in peripheral blood monocytes, the histone deacetylase 8 (HDAC8) plays an important role in increasing chromatin accessibility and thereby in enhancing transcription. Epigenetic modifications in response to BCG vaccination were further suggested by interactions of the 162 upregulated lncRNAs at Visit 2 compared to Visit 1 (Table S8) with the same histone modifications of H3K3me2-3, H2K122ac, and H3T11P identified for upregulated lncRNAs in CD4 T cells (Table 2).

## 4. Discussion

The benefits of the pediatric BCG vaccine are multifold and go far beyond protection against severe complication associated with *Mtb* infection in infants. However, only about 90 years after the introduction of BCG, the mechanisms underlying reduced susceptibility to infectious diseases and lower overall infant mortality after BCG vaccination were discovered. Agnostic immunity of BCG against non-*Mtb* pathogens is mediated through epigenetic modifications in monocytes and their precursors that promote both trained innate immunity and, likely indirectly, heterologous adaptive immunity [14,46]. These altered immune responses in BCG vaccinated individuals are associated with and driven by major metabolomic changes [13,47]. Several studies in human adults have confirmed these findings and deepened our understanding of the interactions between epigenetic modifications, gene regulation and biological pathways important in immunity [46,48,49,50,51,52].

Most of these studies, however, have been conducted in human adults, whereas the primary population receiving the BCG vaccine worldwide are neonates. In 2009, a small study involving five South African infants reported that, 10 weeks after BCG vaccination, genes associated with monocyte activation were upregulated, whereas anti-inflammatory macrophage responses and cell adhesion molecules were suppressed [53]. More recently, consistent with the findings in adults [14], monocytes isolated from Australian infants approximately 1 year after BCG vaccination (n = 63) were reported to have a distinct DNA methylation profile from infants who had not received the vaccine (n = 67) [54]. In the latter study, several differentially methylated sites were found in promoter regions of interferon-stimulated genes. This finding was consistent with data reporting that epigenetic modifications of interferon-stimulated genes are important for trained immunity induction in hematopoietic stem cells [46]. While the current study did not test directly for epigenetic modifications, two histone deacetylases, HDAC8 and HDAC9, were among the top DEGs upregulated in peripheral blood monocytes after BCG vaccination. Furthermore, lncRNAs in both CD4 T cells and monocytes after BCG vaccination were linked to specific histone modifications (see Table 2), including H3K4me3. In BCG-vaccinated adults, the tri-methylation of H3K4 was observed in promoters of several cytokines and Toll-like receptors (TLRs) [55], and histone methylations, specifically H3K4me3 and HeK4me1, are known to be important for activation and macrophage polarization [56].

In addition to enhanced and prolonged innate responses, BCG vaccination has been associated with improved and heterologous T cell immunity. Studies in African infants repeatedly demonstrated that newborns vaccinated with BCG develop potent Th1 and Th17 responses not just to *Mtb* antigens, but also to unrelated pathogens [57,58]. Specifically, the investigators could document increased IFN-γ, TNF-α, and IL-2 production in T cell memory populations, as assessed by flow cytometry after in vitro stimulation. In these studies, mycobacterial-specific and non-specific T cell responses peaked between 6 and 10 weeks following BCG vaccination but persisted for up to 1 year. In human adults, increased IL-17 and IL-22 production was observed up to 1 year after BCG vaccination [59]. The role of T cells was further strengthened by findings that T cell depletion or IFN-γ blockade could abrogate BCG-mediated protective immunity against SARS-CoV-2 challenge or influenza challenge in mice [60,61].

The current study was conducted in Malawian infants and, in contrast to the above-described studies, assessed ex vivo changes in the transcriptome of CD4 T cells collected prior to and 2 to 13 weeks after BCG vaccination *without restimulation* of peripheral blood samples with mycobacterial or heterologous antigens. Control CD4 T cells from unvaccinated infants were not available because all infants in Malawi receive the BCG vaccine. To overcome this limitation, we first defined age-dependent changes in the CD4 T cell transcriptome and then included age at the time of BCG vaccination as a covariate in the analysis of changes in CD4 T cell gene expression after BCG vaccination. Despite the relatively small size of our cohort, we identified numerous genes with altered mRNA expression in CD4 T cells of infants ranging in age from 1 day to 74 days of life. Most pronounced was the upregulation of pathways associated with Th1 and Th17 cell differentiation and TCR signaling. These findings are consistent with the known shift from Th2 to Th1/Th17 immunity as a result of normal immune ontogeny (reviewed in [62,63]. In CD4 T cells collected from infants after BCG vaccination, 33 genes exhibited altered expression applying a log2 FC ≥ 1 and *p* ≤ 0.05. These DEGs were distinct from those DEG induced by age, implying that the 33 DEGs were modulated in response to BCG vaccination. Several of the potentially BCG-induced genes were representative of proteins in metabolic pathways. Among those pathways were glycan and glucagon synthesis, but also the citrate cycle and lipid metabolism. Our results are in agreement with an earlier study documenting changes in the plasma lipid metabolome of Gambian infants within the first seven days after BCG vaccination [15] and with studies that assessed the impact of BCG on glucose and lipid metabolism [15,64]. The ability of BCG to modify metabolomic parameters may not only be important for immunity, but also to support the unique metabolomic needs of infants for growth and development [65].

As with any research, our study has limitations. Our sample size was relatively small. Analysis was restricted to the transcriptome, and limited cell numbers did not allow us to perform functional assays to confirm trained and heterologous immunity in CD4 T cells or monocytes after BCG vaccination. Therefore, the findings of the current study should be repeated in a larger cohort with paired CD4 T cells and monocyte samples, as well as other cell populations (e.g., NK cells, CD8 T cells), utilizing now widely available single-cell technologies and to validate the results with complementary functional assays. A recent study in a large cohort of human adults (n = 325) reported that the transcriptome of CD8 T cells is also significantly altered after BCG vaccination [66]. In the latter study, samples were collected prior to, and 3 months after BCG vaccination and mRNA levels were examined after restimulation of PBMC with LPS. To gain insight into cell type-specific transcriptome changes, cellular deconvolution methods were applied. Consistent with earlier in vitro findings by the same group that monocytes from different donors differ in their potential to develop trained immunity features [67], the results indicated that transcriptome changes in monocytes and CD8 T cells were distinct for high and low trained immunity responders [66]. It would be important to confirm whether BCG vaccinated infants can also be grouped into low and high responders with regard to monocyte trained immunity, and if so, what factors determine this outcome. The current study could not test for high versus low BCG responders in monocytes because the small blood volume, and resulting low cell numbers, required us to pool monocytes from different infants. Furthermore, some studies have reported strain-specific responses to BCG [68,69]. Thus, multiple factors, intrinsic to the vaccinee and extrinsic (e.g., vaccine strain), may impact BCG specific and non-specific responses. Large cohort studies will be necessary to identify such factors. Knowledge gained from such studies could provide novel insights into protective immunity against TB in infants, but also against heterologous immunity against other childhood infectious diseases.

As mentioned above, future studies should also examine epigenetic modifications on infant CD4 T cells and monocytes. Although the evidence for the role of lncRNA in trained immunity is limited, interactions of lncRNAs with transcription factors of histone-modifying enzymes, such as EZH2 and HDAC1, as well as with NF-κB and STAT3, demonstrate that lncRNAs actively regulate the gene expression profile of the immune response genes implicated in the induction of trained immunity [70]. Specifically, lncRNAs are involved in the regulation of multiple signaling pathways, including cytokine signaling, MAPK signaling, and TLR signaling, all pathways with importance in the immune response to *Mtb* [71]. Therefore, despite the study limitations, our overall results are consistent with functional alterations and metabolic shifts observed in human BCG vaccinated infants and adults.

## 5. Conclusions

The importance of understanding the infant’s immune response to vaccination is indispensable for the development of new, improved vaccination strategies. Towards this goal, the results reported here deepen the understanding of molecular changes in CD4 T cells that may explain the improved capacity of the infant immune system to respond to pathogens other than mycobacteria after BCG vaccination.

## Figures and Tables

**Table 1:** Infant age at the time of BCG vaccination and blood collection time points.

Sample ID	Sex	Age at BCG Vaccination	Age at 2nd Visit	Interval
		and 1st Blood Collection	Blood Collection	
TBI017	M	1 day	77 days	76 days
TBI019	F	1 day	42 days	41 days
TBI043	M	1 day	70 days	69 days
TBI032	M	6 days	70 days ^a^	64 days
TBI056	M	7 days	84 days	77 days
TBI000	F	8 days	42 days	34 days
TBI015	F	8 days	77 days	69 days
TBI035	M	8 days	84 days	66 days
TBI054	M	8 days	70 days	62 days
TBI016	F	9 days	77 days	68 days
TBI013	F	10 days	77 days ^a^	67 days
TBI049	M	10 days	105 days	95 days
TBI008	F	11 days	42 days	31 days
TBI023	F	11 days ^a^	70 days	59 days
TBI026	M	12 days	77 days	65 days
TBI036	M	13 days	77 days	64 days
TBI053	M	13 days	77 days	64 days
TBI058	M	13 days	77 days	64 days
TBI048	M	16 days ^a^	63 days	47 days
TBI047	F	18 days	56 days	38 days
TBI004	M	31 days ^a^	42 days	11 days
TBI022	M	35 days	no sample	
TBI025	F	47 days	77 days	30 days
TBI038	F	47 days	77 days	30 days
TBI042	M	60 days	70 days	10 days
TBI024	F	74 days ^a^	112 days	38 days

^a^ no RNA sequencing data.

**Table 2:** Long non-coding RNAs and histone modifications.

Histone	Name	FDR *p* Value		Function
		CD4 T Cells	Monocytes	
H3K4me2-3	histone 3 with demethylation at lysine 2 or 3	3.84 × 10^−15^	8.80 × 10^−13^	transcription activation; recovery of protein synthesis following DNA damage
H3K122ac	histone 3 with acetylation at lysine 122	1.83 × 10^−10^	4.85 × 10^−7^	transcription activation
H3T11P	histone 3 with phosphorylation at threonine 11	1.33 × 10^−6^	0.00025	Response to nutritional stress and metabolic changes

## Data Availability

The transcriptome data are uploaded to the GEO database at NCBI, under accession number GSE288201.

## Supplementary Materials

The following supporting information can be downloaded at https://www.mdpi.com/article/10.3390/vaccines13070706/s1: Table S1: The list of antibodies used for FACS* staining and cell sorting. Table S2: Age-dependent DEGs in CD4 T cells. Table S3: DEGs induced by BCG vaccination in CD4 T cells. Table S4: Interval-dependent DEGs in infant CD4 T cells. Table S5: KEGG pathways associated with DEGs in infant CD4 T cells (NetworkAnalyst). Table S6: Long non-coding RNAs of infant CD4 T cells in response to BCG vaccination. Table S7: DEGs induced in monocytes after BCG vaccination. Table S8: Long non-coding RNAs of infant monocytes in response to BCG vaccination.
