# Supplementary material for: BCG Vaccination Potentially Modulates the Transcriptome of Infant CD4 T Cells in Addition to Age-Dependent Immune Ontogeny-Associated Changes
Source: Vaccines (Basel). 2025 Jun 29;13(7):706. doi: 10.3390/vaccines13070706 (PMC12298075; doi:10.3390/vaccines13070706)
Supplement: Supplementary file 1 [file vaccines-13-00706-s001.zip › Table S1.pdf]

Table S1: The list of antibodies used for FACS* staining and cell sorting

Marker	Fluorochrome*	Clone	Vendor	Volume of Antibody (mcl)
Viability dye	Aqua	NA	Invitrogen	0.5
CD3	BV421	SP34-2	Becton Dickinson (BD)	2.0
CD4	FITC	L200	Becton Dickinson (BD)	2.0
CD14	BV786	M5E2	Becton Dickinson (BD)	2.0
CD16	PE-CF594	3G8	Becton Dickinson (BD)	2.0
CD20	APC-H7	2H7	Becton Dickinson (BD)	2.0
CD56	BB700	B159	Becton Dickinson (BD)	5.0

* FACS, fluorescence-activated cell sorting; NA, not applicable; BV-Brilliant violet, FITC-Fluorescein isothiocyanate, APC-Allophycocyanin, BB-Brilliant blue.
